# IS THERE ANY CHANGE IN PHENOTYPIC CHARACTERISTICS COMPARING 5 TO 10 YEARS OF FOLLOW-UP IN OBESE PATIENTS UNDERGOING ROUX-EN-Y GASTRIC BYPASS?

**DOI:** 10.1590/0102-672020190001e1453

**Published:** 2019-10-21

**Authors:** Carla Barbosa NONINO, Bruno Affonso Parenti de OLIVEIRA, Raoana Cássia Paixão CHAVES, Luciana Tabajara Parreiras e SILVA, Marcela Augusta de Souza PINHEL, Flávia de Campos FERREIRA, Gabriela da Costa ROCHA, Simara Paganini DONADELLI, Julio Sergio MARCHINI, Wilson SALGADO-JUNIOR, Carolina Ferreira NICOLETTI

**Affiliations:** 1 Internal Medicine Department, Ribeirão Preto Medical School, University of São Paulo, Ribeirão Preto, SP, Brazil; 2 Department of Surgery and Anatomy, Ribeirão Preto Medical School, University of São Paulo, Ribeirão Preto, SP, Brazil

**Keywords:** Obesity, Bariatric surgery, Nutritional deficiencies, Weight regain, Obesidade, Cirurgia bariátrica, Deficiencias nutricionais, Reganho de peso

## Abstract

**Background:**

*:* Bariatric surgery promotes significant weight loss and improvement of associated comorbidities; however, nutrients deficiencies and weight regain may occur in the middle-late postoperative period.

**Aim:**

To investigate nutritional status in 10 years follow-up.

**Methods:**

*:* Longitudinal retrospective study in which anthropometric, biochemical indicators and nutritional intake were assessed before and after one, two, three, four, five and ten years of Roux-en Y gastric bypass through analysis of medical records.

**Results:**

*:* After ten years there was a reduction of 29.2% of initial weight; however, 87.1% of patients had significant weight regain. Moreover, there was an increase of incidence of iron (9.2% to 18.5%), vitamin B12 (4.2% to 11.1%) and magnesium deficiency (14.1% to 14.8%). Folic acid concentrations increased and the percentage of individuals with glucose (40.4% to 3.7%), triglycerides (38% to 7.4%), HDL cholesterol (31 % to 7.4%) and uric acid (70.5% to 11.1%) abnormalities reduced. Also, there is a reduction of food intake at first year postoperative. After 10 years, there was an increase in energy, protein and lipid intake, also a reduction in folid acid intake.

**Conclusions:**

*:* Roux-en Y gastric bypass is an effective procedure to promote weight loss and improve comorbidities associated with obesity. However, comparison between postoperative period of five and 10 years showed a high prevalence of minerals deficiency and a significant weight regain, evidencing the need for nutritional follow-up in the postoperative period.

## RESUMO

**Racional:**

A cirurgia bariátrica promove importante perda ponderal e melhora das comorbidades associadas; entretanto, deficiências nutricionais e reganho de peso podem ocorrer no pós-operatório médio e tardio.

**Objetivo:**

Investigar a evolução do estado nutricional de pacientes após cinco e 10 anos de pós-operatório.

**Método:**

Estudo retrospectivo longitudinal, no qual indicadores antropométricos, bioquímicos e a ingestão alimentar foram avaliados no período pré-operatório e após um, dois, três, quatro, cinco e dez anos da operação, por meio de revisão de prontuários.

**Resultados:**

Após 10 anos observou-se redução de 29,2% do peso inicial; no entanto, 87,1% dos pacientes tiveram reganho significativo de peso. Além disso, houve aumento da incidência de deficiência de ferro (9,2% para 18,5%), vitamina B12 (4,2% para 11,1%) e magnésio (14,1% para 14,8%). As concentrações de ácido fólico aumentaram e a porcentagem de indivíduos com alterações na glicemia (40,4% a 3,7%), triglicérides (38% a 7,4%), colesterol HDL (31% a 7,4%) e ácido úrico (70,5% a 11,1%) diminuiu. Além disso, houve redução na ingestão alimentar no primeiro ano de pós-operatório. Após 10 anos, houve aumento na ingestão de energia, proteína e lipídios, e redução na de ácido fólico.

**Conclusões:**

A derivação gástrica em Y-de-Roux é procedimento eficaz para promover perda de peso e melhorar as comorbidades associadas à obesidade. Entretanto, a comparação entre os períodos pós-operatórios de cinco e 10 anos mostrou que uma porcentagem de pacientes apresenta deficiências de vitaminas e minerais e reganho de peso significativo, evidenciando a necessidade do acompanho nutricional no período pós-operatório.

## INTRODUCTION

Obesity is a chronic disease that affects millions of individuals and its incidence has been increased in pandemic proportions^27^. Bariatric surgery is considered the most effective treatment, resulting in significant weight loss, as well as improvement of associated comorbidities^18^ ^,^ ^19^. Considering surgical techniques, Roux-en-Y gastric bypass (RYGB) is the most commonly performed surgical procedure^18^ ^,^ ^19^.

Despite the favorable results associated with bariatric surgery, nutritional deficiencies, due to changes in dietary intake, as well as reduction of gastric capacity and intestinal absorption surface, are very common^10^ ^,^ ^39^. Thus, micronutrient supplementation is recommended to prevent post-surgical deficiencies^10^ ^,^ ^38^. In addition, several long-term studies have identified recovery of weight lost in a portion of patients submitted to the surgery, which can be attributed to the presence of eating disorders, dilation of the gastric pouch, physical inactivity, endocrine and metabolic changes ^2^ ^,^ ^8^ ^,^ ^17^ ^,^ ^21^ ^,^ ^28^ .

Given the importance of evaluating patients submitted to RYGB in the mid-late postoperative period, this study aimed to assess the evolution of nutritional status, including changes in the anthropometric and biochemical profile in a ten years follow-up, contributing to a better understanding of nutritional consequences.

## METHODS

### Patients and study design

All procedures performed were in accordance with the ethical standards of the institutional and/or national research committee and with the 1964 Helsinki declaration and its later amendments or comparable ethical standard. Informed consent was obtained from all individual participants included in the study.

This longitudinal retrospective study enrolled mixed ethnicity obese patients (body mass index - BMI>35 kg/m^2^) who underwent bariatric surgery by RYGB technique between years 2000 and 2009 at the bariatric surgery center of a Brazilian public hospital. Patients who had undergone modification in RYGB, those who died and patients who lost follow-up with the medical team were excluded from the study.

Anthropometric, biochemical and nutritional intake data collection from preoperative and postoperative time (one, two, three, four, five and ten years after surgery) was performed by analyzes of medical records. The study included all patients undergoing RYGB in this service at least ten years after surgery.

### Surgical procedure

Surgery consisted in a reduction of gastric capacity (small gastric portion between 20-50 ml) and a gastric bypass of the duodenum and proximal jejunum with foods and biliary tracts measuring approximately 100 cm. All surgeries were laparotomic performed by the same team of surgeons. All patients used multivitamin-mineral supplementation daily, starting on the 15^th^ day after surgery. According to this recommendation, they ingested one capsule per day of the Maternal^®^ supplement.

### Phenotic characteristics

Anthropometric evaluation was performed with weight, height, BMI, percentage of excess weight loss (%EWL) and weight regain. The %EWL was calculated by the difference between the preoperative weight and the ideal weight (BMI=22 kg/m²). The weight regain (%) was calculated by the percentage difference between the final weight in the postoperative period of ten years and the lowest weight achieved during this postoperative period. A 10% cut was defined to indicate the occurrence of significant recovered weight^16^.

Biochemical parameters [glycemia, total cholesterol (TC), fraction of high density lipoprotein (HDLc), fraction of low density lipoprotein cholesterol (LDLc), triglycerides (TG), hemoglobin, hematocrit, iron, iron binding capacity, magnesium, calcium, vitamin B12, folic acid, total protein, albumin, uric acid, zinc, inorganic phosphorus, urea, creatinine, gamma-glutamyl transpeptidase (gamma-GT), aspartate aminotransferase (AST) and alanine aminotransferase (ALT)] were determined according to tests performed routinely by the Center for Bariatric Surgery.

Dietary intake was evaluated with 24-h food recall and the energy, macronutrient (carbohydrate, protein and lipid) and micronutrients (iron, calcium, folic acid and vitamin B12) intake were estimated through specific software. Also, vomiting and the ease/difficulty of ingesting specific foods were assessed according to patients’ reports (yes or no, objective questions). 

### Statistical analysis

Values were expressed as mean±standard deviation. The linear regression model with mixed effects was used for the longitudinal evaluation of anthropometric and biochemical variables, with SAS^®^ 9.3 software support, using PROC MIXED. It was established as significant p<0.05.

## RESULTS

Until the present moment, 830 bariatric procedures have been performed in referred hospital. Of this number of patients, 441and 110 completed five and 10 years postoperatively, respectively and their medical records were select for the present study. Of them, 82.7% were female and mean age was 44.4±10.4 years. 

According to the anthropometric indicators in the pre- and postoperative period (Table 1), weight reduction was observed until the second year after the operation and maintenance of the values in the following three years. After five years, the total weight loss was 34.3%, corresponding to 59.8% of the excess weight. In fact, 75.3% of the patients reached more than 50% of %EWL in this period. However, despite weight loss by a large percentage of patients, it was observed that 40% had a significant weight gain after five years of RYGB. The classification of nutritional status by BMI showed a reduction in the number of patients with grade III obesity, but only 4.8% reached normal weight status. More interestingly, even though there was a weight loss of 29.2% of preoperative weight after 10 years of RYGB, corresponding to 51.5% of the excess weight, this period was evidenced by the larger number of patients (87.1%) that regained the weight lost.

**TABLE 1:** Evolution of anthropometric indicators at the preoperative time and 1, 2, 3, 4, 5 and 10 years after Roux-en-Y gastric bypass

		Weight (kg)			BMI (kg/m^2^)	
	n	Mean±SD	∆%	n	Mean±SD	∆%
Preoperative	409	134.3±23.5		396	50.5±8.0	
1 year PO	268	90.1±18.4^a^	-33.7	264	33.6±6.3^a^	-33.8
2 years PO	205	85.9±18.0^a,b^	-5.5	204	32.1±5.8^a,b^	-5.4
3 years PO	171	87.0±17.7^a^	1.4	172	32.4±5.7^a^	1.3
4 years PO	130	88.3±18.2^a^	2.3	129	33.0±5.8^a^	2.3
5 years PO	85	88.7±16.7^a^	2.2	83	33.2±5.4^a^	1.8
10 years PO	27	95.4±19.3^a,c^	7.5	27	36.317.0^a,c^	4.1

PO=postoperative time; n=number of patients evaluated; BMI=body mass index; a=p<0.05 compared to preoperative time; b=p<0.05 compared to 1year PO; c=p<0.05 compared to 5year PO; ∆%=percentage of weight loss or regain (negative values represent loss and positive values regain)

Table 2 shows fasting blood glucose and lipid profile before and after surgery. We noted that the major changes occurred in the first postoperative year, with a significant reduction in glycemia and triglycerides and an increase in HDLc concentrations. These changes remained until the 10^th^ year postoperatively. Decreased values of serum and LDLc were observed in the first year after surgery. However, after five years, 23% and 18% of the patients presented increased CT and LDLc, respectively, which remained until the end of the study.

**TABLE 2:** Evolution of glycemia and lipid profile indicators at the preoperative and 1, 2, 3, 4, 5 and 10 years after Roux-en y gastric bypass

		Glycemia			TC			TG			LDLc			HDLc	
		(mg/dl)			(mg/dl)			(mg/dl)			(mg/dl)			(mg/dl)	
	n	Mean±SD	(%)	n	Mean±SD	(%)	n	Mean±SD	(%)	n	Mean±SD	(%)	n	Mean±SD	(%)
Preoperative	166	108.0±38.7	40	360	182.4±40.0	31	357	146.8±73.1	38	351	114.1±33.2	29	358	40.4±10.7	31
1 year PO	161	85.1±10.5^a^	6.2	332	159.9±30.5^a^	9.3	332	86.4±34.6^a^	4.8	325	95.5±25.0^a^	8.9	326	47.2±10.0^a^	8
2 years PO	150	84.5±11.8^a^	6.7	147	165.2±33.8^a^	14	148	80.5±36.0^a^	4.1	146	94.8±26.8^a^	7.5	147	54.2±13.0^a,b^	3.4
3 years PO	136	83.9±9.9^a^	5.1	130	166.6±32.4^a^	12	130	78.5±33.0^a^	4.6	130	98.1±27.1^a^	10	130	53.3±11.4^a,b^	3.8
4 years PO	104	84.6±11.5^a^	5.8	100	174.9±37.8^b,c^	27	100	81.9±38.3^a^	9	101	103.8±29.9^a,b,c^	19	101	54.3±12.6^a,b^	5.9
5 years PO	64	83.9±13.2^a^	4.7	61	175.3±37.0^b^	23	62	75.8±29.8^a^	3.2	62	105.5±29.6^a,b,c^	18	62	54.5±12.1^a,b^	3.2
10 years PO	27	85.1±7.9^a^	3.7	27	164.2±50.2	29.6	27	81.4±36.4^a^	7.4	27	98.1±32.3	29.6	27	54.7±14.9^a^	7.4

PO=postoperative time; n=number of patients evaluated; (%)=percentage of patients with values outside the normal range; TC=total cholesterol total; LDLc=low density lipoprotein; HDLc=high density lipoprotein; TG=triglycerides; a=p<0.05 compared to preoperative; b=p<0.05 compared to one year PO; c=p<0.05 compared to two years PO

Futhermore, serum hemoglobin and hematocrit levels reduced and maintained until the end of the study. Ferritin decreased significantly until the second postoperative year without changes in values after this period. In the first and second postoperative years, an increase was evident in iron concentrations. However, after 10 years, 18.5% and 11.1% of the patients had iron and ferritin deficiency, respectively (Table 3).

**TABLE 3:** Evolution of iron metabolism biochemical indicators at preoperative time and 1, 2, 3, 4, 5 and 10 years after Roux-en-Y gastric bypass

		Iron			LIBC			Ferritin			Hemoglobin			Hematocrit	
		(ug/dl)			(ug/dl)			(ng/ml)			(g/dl)			(%)	
	n	Mean±SD	(%)	n	Mean±SD	(%)	n	Mean±SD	(%)	n	Mean±SD	(%)	n	Mean±SD	(%)
Preoperative	360	74.8±27.6	9.2	346	248.1±66.3	35.8	328	161.4±133.0	1.8	434	13.3±1.7	15.7	441	40.5±4.9	11.6
1 year PO	337	84.3±35.3^a^	8.9	243	218.5±79.3^a^	31.1	315	134.4±133.6^a^	3.5	336	12.9±1.9^a^	28	335	39.5±4.2^a^	16.7
2 years PO	152	83.4±38.6^a^	10.5	133	261.9±96.6^b^	43.8	143	92.1±103.7^a,b^	7	148	12.7±2^a^	31.8	148	39.0±4.4^a^	17.6
3 years PO	137	75.2±35.3^b,c^	17.5	122	273.1±97.3^a,b^	43.8	129	76.2±104.9^a,b^	14.7	139	12.3±2.3^a,b^	34.5	139	37.6±5.5^a,b,c^	24.5
4 years PO	99	80.5±39.2	14.1	89	267.2±90.6^b^	39.4	95	58.7±87.6^a,b,c^	8.4	104	12.4±1.8^a,b^	35.6	104	38.0±3.7^a,b^	19.2
5 years PO	65	72.6±33.4^b,c^	18.5	68	275.2±91.4^a,b^	50	60	40.0±53.5^a,b,c^	13.3	70	12.4±1.4^a,b^	38.6	70	37.4±5.5^a,b,c^	20
10 years PO	27	75.4±49.4^a,e^	18.5	27	243.8±114.8^d,e^	44.4	27	54.1±86.4^a,b,c^	11.1	27	12.7±2.2^a^	22.2	27	38.7±5.9^a,b^	22.2

PO=postoperative time; n=number of patients evaluated; (%)=percentage of patients with values outside the normal range; LIBC=latent iron binding capacity; a=p<0.05 compared to preoperative; b=p<0.05 compared to one year PO; c=p<0.05 compared to two years PO; d=p<0.05 compared to three years PO; e=p<0.05 compared to five years PO

A reduction in total protein and vitamin B12 concentrations was also observed after the first year. Nonetheless, there were no changes in albumin concentrations and an increase in serum folic acid levels. After 10 years of follow-up, 11.1% of the patients had vitamin B12 deficiency (Table 4).

**TABLE 4:** Evolution of vitamin and protein metabolism biochemical indicators at preoperative time and 1, 2, 3, 4, 5 and 10 years of Roux-en-Y gastric bypass

		Albumin			Total protein			Vitamin B12			Folic acid	
		(g/dl)			(g/dl)			(Ng/ml)			(Pg/ml)	
	n	Mean±SD	(%)	n	Mean±SD	(%)	n	Mean±SD	(%)	n	Mean±SD	(%)
Preoperative	380	4.1±1.9	5.8	345	7.0±0.5	3.5	308	408.2±201.0	4.2	315	10.1±5.4	1.3
1 year PO	328	4.2±2.1	4.0	296	6.7±0.5^a^	3.7	294	346.4±202.6^a^	6.8	278	15.1±6.5^a^	0.7
2 years PO	152	4.1±0.3	2.6	148	6.8±0.4^a^	4.1	142	315.1±171.6^a^	12	143	14.9±6.7^a^	0
3 years PO	135	4.4±3.2	1.5	131	6.7±0.5^a^	3.8	122	332.2±189.0^a^	11.5	126	15.0±6.3^a^	0.8
4 years PO	100	4.1±0.2	2	94	6.7±0.5^a^	3.2	94	284.3±178.9^a,b^	20.2	90	15.8±6.8^a^	2.2
5 years PO	58	4.2±0.2	1.7	59	6.7±0.4^a^	1.7	62	310.4±186.0^a^	17.7	62	16.3±6.3^a^	0
10 years PO	27	4±0.4	3.7	27	6.5±0.7^a^	11.1	27	379.8±255.6	11.1	27	18.3±6.5 ^a^	3.7

PO=postoperative time; n=number of patients evaluated; (%)=percentage of patients with values outside the normal range; a=p<0.05 compared to preoperative; b=p<0.05 compared to one year PO; c=p<0.05 compared to two years PO

Table 5 shows the biochemical indicators of mineral metabolism. There was a decrease in serum zinc concentrations up to the second postoperative year, with no significant changes afterwards. Five years after RYGB, serum calcium decreased and after 10 years there was a significant increase, while inorganic phosphorus concentrations remained unchanged. The values of sodium and potassium increased, respectively, for the first and second year of treatment, with maintenance in the following years. Magnesium concentration increased one year after surgery, but was reduced after 10 years. It is important to highlight that ten years after the procedure, the percentage of magnesium deficiency was 14.8%.

**TABLE 5:** Evolution of mineral metabolism biochemical indicators at preoperative time and 1, 2, 3, 4, 5 and 10 years after Roux-en-Y gastric bypass

		Calcium			Sodium			Potassium			Zinc			Magnesium			Inorganic phosphorus	
		(mg/dl)			(meq/l)			(meq/l)			(ug%)			(meq/l)			(mg/dl)	
	n	Mean±SD	(%)	n	Mean±SD	(%)	n	Mean±SD	(%)	n	Mean±SD	(%)	n	Mean±SD	(%)	n	Mean ±SD	(%)
Preoperative	287	9.3±0.8	7.3	405	138.5±7.0	8.4	403	4.2±0.4	3.2	311	88.7±14.7	1.0	320	1.6±0.2	14.1	324	4.4±4.9	0.6
1 year PO	314	9.3±0.6	6.4	334	140.7±3.0^a^	1.2	328	4.2±0.4	1.5	224	83.0±12.6^a^	0.6	319	1.7±0.2^a^	11.3	302	4.2±1.6	0.3
2 years PO	146	9.3±0.7	8.2	152	140.0±3.5^a^	2.0	151	4.3±0.4^a^	1.3	123	80.4±12.0^a, b^	0.7	142	1.6±0.2	8.5	139	4.2±3.0	0
3 years PO	131	9.2±0.7	10.7	139	139.9±2.7^a^	2.2	133	4.3±0.3^a^	0.8	111	80.2±11.0^a,b^	0	124	1.6±0.2	5.6	127	3.9±0.6	0
4 years PO	99	9.2±0.7	9.1	112	139.8±2.6^a^	3.6	102	4.3±0.4^a,b^	0	87	82.7±13.4^a^	1.1	88	1.6±0.2	3.4	90	4.3±3.4	0
5 years PO	59	9.0±0.6^a,b,c^	10.2	77	140.5±3.0^a^	2.6	63	4.3±0.4	1.6	58	82.9±11.3^a^	0	59	1.7±0.2^a,c^	5.1	59	4.5±4.0	0
10 years PO	27	9.2±0.5^d^	7.4	27	139.1±3.2	3.7	27	4.3±0.4	3.7	27	89.3±11.0	0	27	1.5±0.2^b,d^	14.8	27	4.4±3.8	7.4

PO=postoperative time; n=number of patients evaluated; (%)=percentage of patients with values outside the normal range; LIBC=latent iron binding capacity; a=p<0.05 compared to preoperative; b=p<0.05 compared to one year PO; c=p<0.05 compared to two years PO; d=p<0.05 compared to five years PO

Also, in the first postoperative year, there was a reduction in serum levels of uric acid and urea, but there is no change in creatinine levels. Hepatic indicators (gamma-GT, AST and ALT) decreased during the first year after RYGB and up to the 5^th^ postoperative year. The percentage of individuals with changes in serum uric acid, Gamma GT, AST at the end of the study was, respectively, 11.1%, 3.7% and 7.4% (Table 6).

**TABLE 6:** Evolution of liver and kidney metabolism biochemical indicators at preoperative time and 1, 2, 3, 4, 5 and 10 years after Roux-en-Y gastric bypass

	Uric acid			Urea				Creatinine			Gama GT			AST			ALT	
		(mg/dl)			(mg/dl)			(mg/dl)			(mg/dl)			(u/l)			(u/l)	
	n	Mean±SD	(%)	n	Mean±SD	(%)	n	Mean±SD	(%)	n	Mean±SD	(%)	n	Mean±SD	(%)	n	Mean±SD	(%)
Preoperative	371	6.3±1.7	70.5	423	25.9±8.6	1.2	416	0.8±0.2	1.7	365	44.1±33.4	23.4	384	26.1±16.4	18.5	379	33.5±23.2	3.7
1 year PO	326	4.4±1.3^a^	32.4	325	24.7±8.9^a^	1.2	327	0.8±0.4	0.9	313	24.8±14.4^a^	5.8	329	22.9±13.8^a^	2.7	330	23.3±15.8^a^	0
2 years PO	149	4.2±1.2^a^	21.5	150	26.7±8.0^b^	1.3	149	0.8±0.2	0	145	26.4±21.5^a^	8.3	151	23.0±12.0^a^	6.6	151	22.8±11.9^a^	0
3 years PO	130	4.0±1.2^a^	9.2	136	26.4±9.5	2.2	133	0.7±0.1^a^	0	122	26.0±21^.0a^	4.9	131	22.4±9.5^a^	6.9	130	21.5±21.6^a^	0
4 years PO	96	3.9±1.2^a^	10.4	101	26.0±7.7	0	99	0.8±0.1	0	97	24.6±15.0^a^	4.1	100	22.8±7.9^a^	8	101	19.0±9.8^a,b^	0
5 years PO	59	3.9±1.2^a,b^	13.6	62	25.0±9.4	1.6	61	0.9±0.8^d^	1.6	60	26.0±24.0^a^	6.7	63	22.3±5.3^a^	6.3	63	18.5±9.0^a^	0
10 years PO	27	4.2±1.4^a^	11.1	27	27.9±9.4	0	27	0.7±0.1^a^	0	27	25.6±4.6^a^	3.7	27	25.0±10.7	7.4	27	20.2±8.0^a^	0

PO=postoperative time; n=number of patients evaluated; (%)=percentage of patients with values outside the normal range; Gama-GT=gamma-glutamyl transpeptidase; AST=aspartate aminotransferase; ALT=alanine aminotransferase; a=p<0.05 compared to preoperative; b=p<0.05 compared to one year PO; c=p<0.05 compared to two year PO; d=p<0.05 compared to three years PO

A reduction in the consumption of calories, macronutrients, iron, vitamin B12 and folic acid were observed from at first year postoperative, with no difference until five years of follow-up. Calcium intake increased at 1^st^ year after surgery, and decrease after the second, thus maintaining this intake at five years postoperative time (Table 7). After 10 years, there was an increase in energy, protein and lipid intake, also a reduction in folid acid intake.

**TABLE 7:** Evolution of food intake at preoperative time and 1, 2, 3, 4, 5 and 10 years after Roux-en-Y gastric bypass

	n	Energy (kcal/day)	Protein (g/day)	Carbohydrate (g/day)	Lipid (g/day)	Calcium (mg/day)	Iron (mg/day)	Vitamin B12 (mg/day)	Folic acid (mg/day)
Preoperative	72	1505.6±582.7	73.3±27.5	200.9±90.2	47.4±21.9	495.7±227.6	12.6±5.9	3.5±2.1	297.7±146.9
1 year PO	72	965.2±274.6^a^	43.6±13.6^a^	131.4±51.2^a^	30.8±11.0^a^	596.8±248.5^a^	6.5±1.7^a^	2.2±1.3^a^	218.3±121.4^a^
2 years PO	72	1005.8±365.4^a^	46.0±16.5^a^	133.6±56.5^a^	33.3±14.9^a^	487.8±258.8^b^	6.9±2.7^a^	2.1±1.2^a^	184.9±109.6^a^
3 years PO	72	981.4±336.6^a^	45.2±18.8^a^	126.2±40.4^a^	34.1±16.5^a^	480.1±258.7^b^	7.0±2.9^a^	4.9±10.5^a,b,c^	202.9±116.2^a^
4 years PO	72	949.3±343.2^a^	46.1±15.0^a^	122.9±54.0^a^	31.8±15.2^a^	421.8±205.9^b^	7.1±2.5^a^	2.7±3.6^a,d,e^	193.7±85.9^a^
5 years PO	72	1014.2±338.4^a^	44.8±20.3^a^	130.4±45.6^a^	37.7±15.4^a^	465.8±286.0^b^	6.8±2.2^a^	2.0±1.3^a,e^	186.8±110.5^a^
10 years PO	27	1300.9±581.0^b,c,d,e^	59.7±26.1^b^	164.8±90.7	44.8±23.1^b,c,d,e^	528.1±355.5^e,f^	8.3±4.4	3±2.6^f^	109.7±85.9^a,b,c,d,e,f^

PO=postoperative time; n=number of patients evaluated; a=p<0.05 compared to preoperative; b=p<0.05 compared to one year PO; c=p<0.05 compared to two years PO; d=p<0.05 compared to three years PO; e=p<0.05 compared to four years PO; f=p<0.05 compared to five years PO

After five years of RYGB, vomiting was reported by 69% of the patients. The occurrence of these was associated with specific food intake by 63% of the patients, mainly meat (65%) and rice (41%). Only 15% of patients reported accepting all types of food well. It was observed a better tolerance to white meat (77%) compared to red meat (41%) and better tolerance to bread (87%) and pasta (69%) compared to rice (56%). About 20% of the patients reported intolerance to leafy vegetables and 31% to sweet. After 10 years, it was observed that the great majority of the patients reported ease intake of vegetables, and more than 80% reported being able to eat bread; however, about 40% reported difficulty eating red meat.

## DISCUSSION

This study evaluates the impact of bariatric surgery on the nutritional phenotypic characteristics of obese individuals in the medium to late postoperative period characterized by 10 years follow-up. Despite significant weight loss until the second postoperative period, we observed that the majority of patient recovered the weight lost after 10 years of RYGB. Surgical treatment led to the improvement of biochemical indicators, such as lipid profile and blood glucose, and a reduction in the number of patients with inadequate levels of liver enzymes and uric acid. However, surgery has also resulted in an increased of vitamins and mineral deficient prevalence such as iron and vitamin B12.

In this study, after two years of RYGB there was a 39.2% reduction in initial weight, a result that is similar to the literature^1^ ^,^ ^3^ ^,^ ^9^ ^,^ ^20^. In fact, treatment success can be evaluated by %EWL, which should correspond to 50-75% of the excess weight in the preoperative period. Our results show that %EWL of 59.8% and 51.5%, at five and 10 years postoperative respectively, considered a successful treatment ^1^ ^,^ ^9^ ^,^ ^11^ ^,^ ^22^ ^,^ ^36^.

However, weight regain started as soon after the second postoperative year, as observed in literature studies^12^ ^,^ ^20^ ^,^ ^37^. Freire et al.^12^ during the postoperative period of two to five years, showed a significant weight recover in 24.2% of patients submitted to RYGB, similar to the data found by Bastos et al.^3^ and Nicoletti et al.^28^ over the same period. However, we emphasize that our results show a higher percentage of patients (40%) with the weight regain in this postoperative period.

The evaluation of the biochemical profile showed improvement in serum glucose, with 93.8% of patients with normal levels after one year of procedure. Carvalho et al.^6^ observed that after one year, 42.5% of the patients had normal concentrations of fasting glucose. In this study, surgery led to an improvement in lipid profile with decrease in the number of patients with dyslipidemia, which is similar to the literature data^1^ ^,^ ^5^ ^,^ ^7^. Previous published data showed that the improvement in blood glucose and lipid profile occurs mainly in the 1^st^ postoperative period^16^, which can be attributed to the increase in hepatic insulin sensitivity observed after weight loss after surgery^1^ ^,^ ^7^.

Moreover, studies have shown reduced hepatic steatosis following bariatric surgery. The possible mechanisms responsible for reducing the incidence of non-alcoholic fatty liver disease in these patients correspond to improved peripheral insulin sensitivity and reduced inflammatory markers^7^ ^,^ ^13^ ^,^ ^14^. Although biochemical concentrations of liver enzymes and their alterations are not considered gold standard for diagnosis and evaluation of hepatic steatosis, many studies use AST and ALT transaminases to evaluate the effect of bariatric surgery on hepatic metabolism. There is a transient elevation of liver enzymes, which tend to remain high for a period of two to six months after surgery. A significant decrease followed this increase in the period from 10 to 12 months after surgery, maintenance of low levels up to 10 years of surgery ^13^ ^,^ ^14^. Thus, our research indicated compliance with the literature data.

Elevated concentrations of serum uric acid are often observed in patients with severe obesity. Several potential mechanisms may explain these cases of hyperuricemia, including increased insulin resistance, which has the opposite effect on renal clearance of uric acid. As in our study, other authors also observed that weight loss induced by RYGB was accompanied by a reduction in uric acid levels and a reduction in the prevalence of hyperuricemia. This effect may result from normalization of hyperinsulinemia and insulin resistance observed after bariatric surgery^33^.

Vitamin B12 deficiency is more related after RYGB. Factors that may contribute to this deficiency include gastric achloridia, reduced food intake, especially red meat, decreased secretion of intrinsic factor and bacterial overgrowth in the intestinal ileum^4^ ^,^ ^5^. In the present study, we observed a reduction in serum concentrations and an increase in the incidence of deficiency. This result is lower than other studies in that the same period, prevalence ranged from 19-35%^35^. The guidelines on vitamin B12 supplementation after bariatric surgery recommend daily doses of 1,000 mg orally or monthly of 10 mg intramuscularly to increase serum concentrations of this vitamin and prevent disability^24^.

After bariatric surgery, the prevalence of patients with folic acid deficiency is 9-38%^35^. In this study, the number of patients with this nutrient deficiency decreased after five years, no patient was deficient and the values observed described below in the literature^35^. The improvement of folic acid levels after bariatric surgery can be explained by the daily supplementation with adaptive mechanisms that allow this absorption of vitamin through the intestine ^4^ ^,^ ^9^ ^,^ ^35^. However, in the period of five to 10 years the prevalence of patients with folic acid deficiency increased to 3.7%. This can be attributed to segment loss, lack of nutritional monitoring, and resistance to changes in eating habits after surgery^32^.

Iron deficiency is the most common cause of anemia in patients undergoing the bariatric procedure. The main factors responsible for the deficit of this mineral correspond to intolerance and aversion to foods with high iron bioavailability, reduction of the production of hydrochloric acid in the stomach and malabsorption due to exclusion of the primary absorption sites^4^ ^,^ ^7^ ^,^ ^9^ ^,^ ^31^. The deficiency of this mineral is observed in 17-45% of patients two and five years after the procedure, respectively^35^. Our results are similar to the Moizé et al.^26^ in which there was a deficiency in 15.5% of patients after five years of surgery. This deficiency was maintained until the tenth year after the procedure.

The impact of bariatric surgery on calcium metabolism is not yet fully understood^9^, but possible contributors to the deficiency correspond to food intolerance to the main sources of this mineral, vitamin D deficiency and exclusion of intestinal areas where this mineral is preferentially absorbed ^4^ ^,^ ^9^. In our study, after five years of RYGB, approximately 10% of patients with calcium deficiency were observed, a result similar to that found by Stein et al.^35^, but higher than that found by Moizé et al.^26^ in the same period. Over the study period, there was a decrease in the percentage of patients with magnesium deficiency. And in the preoperative period, 14.1% presented a deficit, lower than the value observed in other studies in which the prevalence ranged from 19-35%^29^ ^,^ ^35^. After five years of surgery, the percentage of individuals with disabilities was similar to that observed by Moizé et al.^26^.

Protein deficiency is often reported, and mainly observed in combined operations^4^. After RYGB, the deficiency ranges from 3-18% of patients^4^ ^,^ ^35^. In the present study, albumin deficiency decreased over the study period, a result similar to that found by other authors^26^. It should be noted that the patients in this study are systematically followed by a multi-professional team and are advised to take vitamin and mineral supplements daily.

Low food intake was observed in the preoperative period. According to the protocol of preoperative guidelines of the referred hospital, the candidate patients must lose at least 10% of the initial weight before being submitted to surgery. Therefore, the results of the preoperative period may reflect a hypocaloric diet for weight loss^30^. Indeed, the relevant fact was the increase in energy, protein and lipid intake at 10-year postoperative period. This fact may be related to weight regain evidenced in the present study. Moreover, we have to add that problems with binge eating/disorder are quite common among obese patients, including bariatric surgery candidates. Thus, it is important to identify this kind of disorder in bariatric surgery patients and appropriate interventions need to be made at postoperative time aiming maximize weight loss outcomes^25^.

It is important to emphasize as a limitation of the present study the loss of follow-up with the consequent reduced number of patients evaluated over time, as was also observed in other studies^15^ ^,^ ^23^. However, this fact is already expected in longitudinal studies with clinical follow-up and, unfortunately, can not be controlled.

Phenotypic characteristics assessment of patients in the late postoperative period is still of great importance in clinical practice. As observed, there are a large number of patients who recover their weight lost, which may also be associated with the return of comorbidities, worsening of quality of life and greater health expenditure. The loss of follow-up by large numbers of patients is a factor that must be studied. The reasons why patients fail to return to medical appointments are numerous and should be investigated. This loss of follow-up may be one of the determinant factors that lead to weight regain. Genetic and psychological aspects can also be pointed out as predictors of weight regain. The constant monitoring of these patients, the adequacy of nutritional recommendations and the identification of biomarkers for weight changes should be seen as new targets of study and is the major focus of individualized bariatric surgery management.

## CONCLUSION

Roux-en-Y gastric bypass is an effective procedure to promote weight loss and improve comorbidities associated with obesity. The comparison between postoperative period of five and 10 years shows the increase in the percentage of patients who had weight gain, as well as those with mineral, folic acid and protein’s deficiency, emphasizing the need for nutritional monitoring in the postoperative period. 
